# Ethnic Disparities in the Management of Pediatric Subcutaneous Abscesses

**DOI:** 10.3390/children9101428

**Published:** 2022-09-21

**Authors:** Rachael A. Clark, Isabel C. Garcia, Jillian C. Jacobson, Dai H. Chung

**Affiliations:** Department of Surgery, Division of Pediatric Surgery, University of Texas Southwestern Medical Center, Dallas, TX 75235, USA

**Keywords:** subcutaneous, abscess, disparities, race, ethnicity, pediatric, sedation

## Abstract

(1) Background: Significant racial and ethnic disparities affect access to pediatric Emergency Department (ED) and surgical care across the United States. The present study sought to assess the role of racial and ethnic disparities in the management of pediatric subcutaneous abscesses. (2) Methods: A retrospective chart review was performed including ED visits for subcutaneous abscesses in patients < 18 years of age, over a 12-month period. The effects of self-reported ethnicity (Hispanic versus non-Hispanic) and race (Hispanic, Black, Caucasian and Asian) on the diagnosis and management of subcutaneous abscesses were analyzed. (3) Results: 192 patients were identified with an average age of 4.7 ± 5.3 years and 43.8% identified as Hispanic. Non-Hispanic patients were significantly more likely to receive treatment of their SSTI prior to the ED and to be admitted, compared to Hispanic patients. There was no difference in bedside versus operating room incision and drainage (I&D); however, significantly more non-Hispanic patients received procedural sedation for bedside I&D compared to Hispanic patients. There were no differences in outcomes such as recurrence or re-admission based on ethnicity or race. (4) Conclusions: Ethnic and racial disparities exist in the management of subcutaneous abscesses in the United States. Further studies are needed to address the systemic causes of these disparities such as access to tertiary healthcare facilities and systems-based analyses of unconscious bias in healthcare.

## 1. Introduction

Racial and ethnic disparities in healthcare are well documented in the adult population as well as the pediatric population, particularly regarding access to pediatric surgical care across the United States [1]. While additional socioeconomic factors, such as insurance status and a primary language other than English, are associated with decreased access to surgical interventions, there are well perceived racial and ethnic disparities among pediatric surgical patients in the ambulatory setting, independent of income and insurance status [2]. A 2013 study found that Black patients had a significantly longer length of hospital stay and increased costs, and both Black and Hispanic patients had increased post-operative mortality compared to White patients [3], suggesting that similar disparities exist in the inpatient setting.

A large contributor to healthcare disparities is access, or lack thereof, to primary care providers. Mudd et al. demonstrated that decreased access to primary care providers led to an increase in unnecessary hospitalizations and Emergency Department (ED) visits [4]. In addition, the authors found that Black and Hispanic children had significantly less access to primary care compared to White children and that non-White children had the highest risk of ED visits [4].

In addition to variations in access to primary and surgical care, significant racial and ethnic disparities exist in the management of patients in the ED. Previous studies have shown that Black patients were less likely to receive any analgesia or opioids for severe pain when compared to White patients [5,6]. Both non-Hispanic Black and Hispanic children were less likely to receive antibiotics for acute respiratory tract infections in the ED, when compared to non-Hispanic White children [7].

The ED and Pediatric Surgery Departments work together closely for a variety of patient conditions including skin and soft-tissue infections (SSTIs). SSTIs, including cellulitis and abscesses, are a relatively common diagnosis in pediatric patients, and have been increasing in prevalence, largely due to the increase in Methicillin-resistant *Staphylococcus aureus*. The increase in skin and soft tissue infections (SSTI) including abscesses has led to a subsequent increase in incision and drainage (I&D) procedures [8,9]. We have previously shown that the diagnosis of pediatric subcutaneous abscesses is largely clinical and that management varies widely [10]. In a study by Singer et al. evaluating patient versus provider assessment of pain, I&D was considered the second most painful procedure performed in the ED [11]. Despite this, the use of procedural sedation for I&D varies widely [12]. Given previous studies demonstrating disparities in clinical management based on race and ethnicity, the aim of the present study was to evaluate whether racial and ethnic disparities play a role in the management of subcutaneous abscesses in pediatric patients, particularly in relation to procedural sedation.

## 2. Materials and Methods

### 2.1. Study Design

A single-center, retrospective chart review was performed after Institutional Review Board (IRB#2020-0065) approval. Encounters for patients between the ages of two months and 18 years presenting with a subcutaneous gluteal/buttock SSTI to the Children’s Medical Center (Dallas, TX, USA) Emergency Department (ED) from 1 January 2019 to 31 December 2019 were identified and included. ICD-9-CM and ICD-10-CM codes were used to identify the patients. Only gluteal and buttock SSTIs were included to control for potential variations in management due to body site. Exclusion criteria included immunocompromised patients, patients with complications from prior wounds/surgeries, patients with inflammatory bowel disease and patients with asymptomatic or incidentally identified abscesses.

### 2.2. Clinical Factors

Data collected included demographics, past medical history, history of prior treatment or intervention, presenting symptoms, physical exam findings, vital signs, laboratory values, imaging results, procedures performed, medications, and outcomes. Demographics included self-reported race/ethnicity (Hispanic, Asian, Black, Caucasian or other). Examination findings included presence or abscess of cellulitis and/or fluctuance. Medications included oral antibiotics, intravenous (IV) antibiotics, and medications for procedural sedation and anxiolysis. Our exposure of interest was the patient’s self-reported ethnicity and included Hispanic vs. non-Hispanic. We further investigated patient self-reported race including Hispanic, Asian, Black, Caucasian and other.

### 2.3. Outcomes

The primary outcome of interest was use of procedural sedation and/or anxiolysis (PSA) for bedside I&D. A patient was considered to have received PSA if they received ketamine, nitrous oxide or midazolam. Secondary outcomes included admission to the hospital, treatment with intravenous (IV) antibiotics, abscess recurrence, and follow-up. Recurrence was defined as a return to the ED or pediatrician for an abscess in the same location within 30 days of hospital discharge.

### 2.4. Statistical Analysis

Continuous variables are reported as mean ± standard deviation (SD) and categorical variables are reported as N (%). Bivariate analysis of continuous variables was performed using Student’s *t*-test or ANOVAs, as appropriate. Categorical variables were analyzed using chi-square analysis. Data analysis was performed using IBM SPSS (version 28, 2021, Armonk, NY, USA), Statistical significance was determined at a *p* value < 0.05.

## 3. Results

### 3.1. Demographics

**Figure 1:**
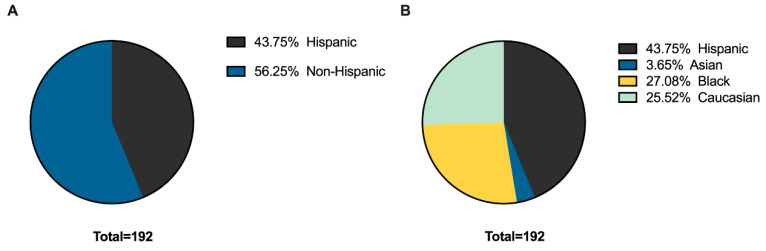
Self-reported (**A**) ethnicity and (**B**) race.

A total of 201 patients between the ages of 2 months and 18 years presenting with a buttock/gluteal SSTI from January 2019 to December 2019 were identified. Out of 201 patients, nine patients met exclusion criteria, resulting in 192 patients total. Demographic and clinical characteristics are summarized in Table 1. The average age was 4.7 ± 5.3 years and 86 (44.8%) of patients were male. A majority of patients identified their race/ethnicity as Hispanic (43.8%), whereas 3.7% identified as Asian, 27.1% identified as Black and 25.5% identified as Caucasian (Figure 1A,B).

### 3.2. Presentation and Evaluation

Over half of the patients reported a subjective fever, *n* = 98 (51.0%). There was no significant difference in past medical history, including history of abscesses, personal and/or family history of MRSA, or co-morbidities between Hispanic and non-Hispanic patients (Table 2). However, only 23 (27.4%) Hispanic patients received treatment for their SSTI from a primary care or non-emergency medicine provider prior to presentation at the ED compared to 50 (46.3%) non-Hispanic patients (*p* < 0.01) (Table 2). A total of 22.6% of Hispanic patients received a trial of antibiotic management prior to ED evaluation compared to 34.3% of non-Hispanic patients; however, this was not a statistically significant difference (*p* = 0.08). There were no significant differences in presenting symptoms, imaging findings, vital signs, laboratory values or physical exam findings such as cellulitis, fluctuance or the area of the abscess, based on ethnicity (Hispanic versus non-Hispanic) (Table 2) or race (Supplemental Table S1).

### 3.3. Primary Outcome

**Figure 2:**
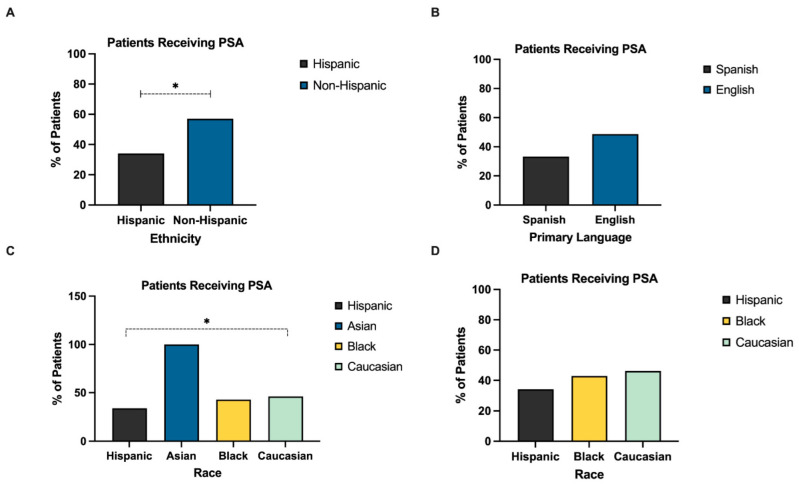
Factors affecting use of procedural sedation and/or anxiolysis. (**A**) Significantly more non-Hispanic patients received PSA compared to Hispanic patients. (**B**) There was no statistically significant difference in PSA between Spanish and English speakers. (**C**) There was a statistically significant difference in PSA based on race, with 46.2% of Caucasian patients, 42.9% of Black patients and 100% of Asian patients receiving PSA, compared to 34.1% of Hispanic patients. (**D**) Comparison of PSA based on race, excluding Asian patients, demonstrates no significant difference in PSA based on race. PSA, procedural sedation and/or anxiolysis. (* = *p* < 0.05).

A total of 78 patients received a bedside I&D. PSA was performed in 43 patients (22.4%). While there was no significant difference in bedside I&D versus I&D in the operating room (OR) based on ethnicity or race, there was a significant difference in patients who received PSA based on both ethnicity and race. As shown in Figure 2A, non-Hispanic patients were significantly more likely to receive PSA compared to Hispanic patients (57.1% vs. 34.1%, *p* < 0.05). To further elucidate the difference in PSA in Hispanic vs. Non-Hispanic patients, the difference in PSA in English speaking versus non-English speaking patients was evaluated. A total of 48.7% of English-speaking patients received PSA compared to 33.3% of non-English speaking patients; however, there was no statistically significant difference in PSA based on primary language (*p* = 0.27) (Figure 2B).

As a cohort, there was a statistically significant difference in rate of PSA based on race (Figure 2C). However, because all three Asian patients who underwent bedside I&D received PSA, an analysis was performed comparing only Hispanic, Black and Caucasian patients, which found that only 34.1% of Hispanic patients received PSA, compared to 42.9% of Black and 46.2% of Caucasian patient. However, this did not reach statistical significance (*p* = 0.06) (Figure 2D).

### 3.4. Secondary Outcomes

Of the 192 patients with SSTIs, 54 (28.1%) were admitted to the hospital and 68 (35.4%) received IV antibiotics. Despite no difference in presenting symptoms, vital signs, imaging findings, abscess size, or laboratory values, significantly more non-Hispanic patients were admitted compared to Hispanic patients (33.3% vs. 21.4%, *p* < 0.05). There was no statistically significant difference in admission rate (*p* = 0.20) or administration of IV antibiotics (*p* = 0.06) based on race (Supplemental Table S1). 

**Figure 3:**
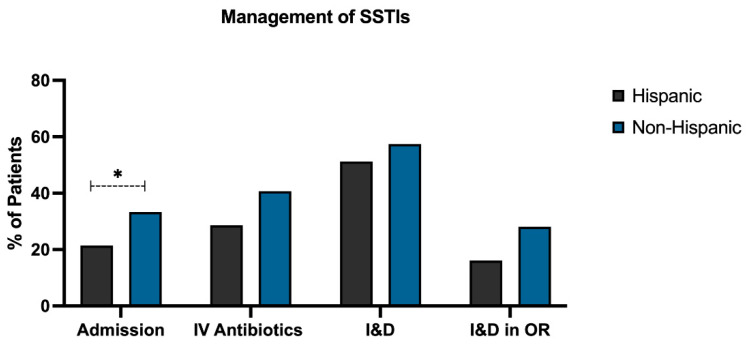
Variations in management of SSTIs based on ethnicity. Significantly more non-Hispanic patients were admitted (33.3%) compared to Hispanic patients (21.4%). Over forty percent of non-Hispanic patients received IV antibiotics (40.7%), while 28.6% of Hispanic patients received IV antibiotics. An I&D was performed in 51.2% of Hispanic patients and 57.4% of non-Hispanic patients. Of patients requiring an I&D, 28.1% of non-Hispanic patients received an I&D in the OR compared to 16.1% of Hispanic patients. SSTI, skin and soft-tissue infection; IV, intravenous; I&D, incision and drainage; OR, operating room. (* = *p* < 0.05).

I&D was performed on 105 (54.7%) patients. There was no significant difference in rate of I&D based on ethnicity (*p* = 0.39) (Figure 2) or race (*p* = 0.73) (Supplemental Table S1). Twenty-seven (25.7%) patients underwent I&D in the operating room (OR) compared to 74.3% who received a bedside I&D. A total of 28.1% of non-Hispanic patients received an I&D in the OR compared to 16.1% of Hispanic patients. However, this was not statistically significant (*p* = 0.30) (Figure 3).

Overall, 15.9% of patients followed up with a provider in clinic (primary care or surgery) and recurrence occurred in 11 patients (5.7%). There was no significant difference in follow-up (*p* = 0.60) or recurrence (*p* = 0.30) between Hispanic and non-Hispanic patients.

## 4. Discussion

Significant ethnic and racial disparities exist in the management of pediatric subcutaneous abscesses. In the present study, we sought to determine the effect of race and ethnicity on the management of SSTIs, including management, such as the need for I&D, and the use of PSA. While there was no difference in need or location of I&D (bedside versus OR), non-Hispanic patients were significantly more likely to receive PSA compared to Hispanic patients. In addition, Hispanic patients were significantly less likely to be admitted, compared to non-Hispanic patients. Although there was no difference in long-term outcomes such as recurrence, re-admission or return to ED based on ethnicity or race, inadequate pain control during I&D can have long-lasting effects, including an enhanced pain experience during subsequent procedures [12]. The differential management of children with SSTI based on ethnicity and race contributes to racial disparities and awareness and active changes in practice are necessary.

The effects of race and ethnicity on I&D and the use of procedural sedation for bedside I&D were examined. We found that non-Hispanic patients were 1.6 times more likely to receive PSA compared to Hispanic patients. A previous study by Uspal et al. in 2015 [12] found that the use of PSA was negatively associated with increasing age and that Black children were 1.4 times less likely the receive sedation. However, we did not find a statistically significant difference in PSA based on race, similar to findings by Chumpitazi et al. in 2017 [9].

Given the significant difference between the use of PSA in Hispanic and non-Hispanic children, we sought to determine the effects of primary language on PSA. While we did not find a significant difference between PSA use in English versus Spanish speaking families, previous studies have demonstrated that Hispanic children received 30% fewer opioids than non-Hispanic children for post-operative pain and that children of parents with Low English Proficiency (LEP) receive fewer pain assessments and are less likely to receive opioids compared to children of proficient English speakers with similar levels of documented pain [6,13,14,15,16]. We may not have seen a significant difference in PSA based on primary language given our institution’s readily accessible in-person medical interpreters, as well as remote interpretation accessible by telephone and video.

We found that non-Hispanic patients were significantly more likely to be admitted to the hospital from the ED compared to Hispanic patients. These findings are consistent with a systematic review that found that Caucasian patients were more likely to be admitted from the ED compared to Black or Hispanic patients [7]. The present study also found that significantly more non-Hispanic patients received treatment prior to the ED for their SSTI compared to Hispanic patients, which may reflect differences in access or utilization of primary care. In addition, Caucasian and Asian patients were significantly more likely to receive a trial of oral antibiotics prior to ED visit compared to Hispanic and Black patients. These findings are consistent with a study by Mudd et al. [4] that demonstrated ethnic and racial disparities in access to primary care. The authors found that the risk of ED visits was highest among non-White patients and that differential access to primary care is a major driver of racial inequities. In addition, it has been shown that delays in care based on ethnicity or race could be secondary to a prior experience of perceived discrimination, which can contribute to delays in timely treatment and underutilization of health care [17].

There are several limitations to the present study including the retrospective nature of the study and the subsequent inability to assess why a patient may or may not have received PSA, such as inadequate staffing or patient/parent refusal, among others. Future studies are needed to address potential differences in documentation and perception of pain scores. In addition, while 192 is a relatively large sample size, there were only seven Asian patients included in this study. These findings may be limited to the present patient population given the increased predominance of Hispanic patients at our institution. However, our large Hispanic patient population may highlight ethnic disparities that did not have reached statistical significance in other studies due to underrepresentation. Future studies are needed to evaluate the racial and ethnic disparities in the management of SSTIs and to assess potential factors contributing to racial and ethnic disparities such as implicit and explicit provider bias.

In conclusion, racial and ethnic disparities exist in the management of SSTIs. Hispanic patients demonstrated decreased rates of primary care treatment prior to ED visits compared to non-Hispanic patients. In the ED, Hispanic patients are significantly less likely to receive PSA compared to non-Hispanic patients, independent of primary language, and are less likely to be admitted to the hospital, despite no difference in laboratory or clinical variables. While there were no statistically significant differences in outcomes such as recurrence, re-admission or return to ED, there are long-term adverse consequences of inadequate pain control and perceived discrimination can lead to delay in timely treatment due to mistrust of healthcare providers [17]. The analysis of potential biases and an improved understanding of individual and institutional factors that impact the management of SSTIs, including the use of PSA, is paramount in order to alleviate the immediate and long-term effects of racial and ethnic disparities among pediatric patients.

## Figures and Tables

**Table 1:** Demographics and patient characteristics.

Variable		All (*n* = 192)	Variable		All (*n* = 192)
Age (years)		4.7 ± 5.3	Subjective Fever		98 (51.0%)
Gender	Male	86 (44.8%)	Exam Findings	Fluctuance	80 (41.7%)
Ethnicity	Hispanic	84 (43.8%)		Cellulitis	106 (53.5%)
Race	Hispanic	84 (43.8%	Ultrasound Performed		104 (54.2%)
	Asian	7 (3.7%)	Temperature (°C)		37.2 ± 0.8
	Black	52 (27.1%)	Heart rate (beats/minute)		123.5 ± 25.5
	Caucasian	49 (25.5%)	Respiratory Rate (breaths/minute)		25.4 ± 5.2
Prior Treatment		73 (38.0%)	WBC count (10^9^ cells/L)		17.7 ± 6.8
Prior Antibiotic Use		56 (29.2%)	CRP level (mg/L)		6.4 ± 5.1
History of Abscess		55 (28.6%)	Area of Abscess (cm^2^)		24.9 ± 35.6
Co-morbidities		10 (5.2%)	Recurrence		11 (5.7%)

WBC, white blood cell count; CRP, c-reactive protein.

**Table 2:** Hispanic vs. Non-Hispanic.

Variable		Hispanic (*n* = 84)	Non-Hispanic (*n* = 108)	*p* Value
Age (years)		5.4 ± 5.8	4.1 ± 4.8	0.08
Gender	Male	41 (47.7%)	45 (41.7%)	0.20
Prior Treatment		23 (27.4%)	50 (46.3%)	0.007
Prior Antibiotic Use		19 (22.6%)	37 (34.3%)	0.08
History of Abscess		25 (45.5%)	30 (44.1%)	0.88
Co-morbidities		2 (2.4%)	8 (7.4%)	0.12
Subjective Fever		44 (52.4%)	54 (50.0%)	0.74
Exam Findings	Fluctuance	31 (36.9%)	49 (45.4%)	0.14
	Cellulitis	49 (58.3%)	57 (52.8%)	0.55
Ultrasound Performed		45 (53.6%)	59 (64.6%)	0.88
Temperature (°C)		37.1 ± 0.8	37.2 ± 0.8	0.42
Heart rate (beats/minute)		121.9 ± 25.6	124.8 ± 25.4	0.45
Respiratory Rate (breaths/minute)		25.1 ± 5.1	25.8 ± 5.3	0.42
WBC count (10^9^ cells/L)		17.0 ± 6.6	18.1 ± 7.0	0.55
CRP level (mg/L)		7.2 ± 5.4	6.0 ± 5.1	0.58
Area of Abscess (cm^2^)		24.2 ± 35.2	25.5 ± 36.0	0.84

WBC, white blood cell count; CRP, c-reactive protein.

## Data Availability

The data presented in this study are available on request from the corresponding author. The data are not publicly available due to privacy.

## Supplementary Materials

The following supporting information can be downloaded at: https://www.mdpi.com/article/10.3390/children9101428/s1, Table S1: Management of SSTIs based on race.
