# Supplementary material for: Ethnic Disparities in the Management of Pediatric Subcutaneous Abscesses
Source: Children (Basel). 2022 Sep 21;9(10):1428. doi: 10.3390/children9101428 (PMC9600215; doi:10.3390/children9101428)
Supplement: Supplementary file 1 [file children-09-01428-s001.zip › children-1906859-supplementary.pdf]

Supplemental Table S1 Management of SSTIs based on race

Variable .	All (n=192)		Hispanic (n=84)		Asian (n=7)		Black (n=52)		Caucasian (n=49)		
	Mean	SD	Mean	SD	Mean	SD	Mean	SD	Mean	SD	*p*-value
Age (years)	4.7	5.3	5.4	5.8	4.9	5.2	4.4	5.2	3.7	4.5	0.30
Area of Abscess (cm²)	24.9	35.6	24.2	35.2	20.0	5.7	26.8	43.7	24.5	28.3	0.93
WBC count (10^9 cells/L)	17.7	6.8	17.0	6.6	17.4	5.3	20.0	7.4	17.1	7.2	0.64
CRP level (mg/L)	6.4	5.1	7.2	5.4	1.9	1.5	9.4	4.5	6.4	5.4	0.20
Temperature (°C)	37.2	0.8	37.1	0.8	36.8	0.4	37.3	0.8	37.2	0.9	0.47
Heart rate (beats/minute)	123.5	25.5	121.9	25.6	120.0	30.3	124.3	26.4	126.0	24.0	0.82
Respiratory Rate (breaths/minute)	25.4	5.2	25.1	5.1	23.4	2.5	25.5	5.4	26.2	5.5	0.47
	N	%	N	%	N	%	N	%	N	%	
Gender (male)	86	44.8%	41	48.8%	2	28.5%	22	42.3%	21	42.9%	0.68
Previous Oral Antibiotics	56	29.2%	19	22.6%	3	42.9%	10	19.2%	24	49.0%	<0.01
History of prior abscesses	55	28.6%	25	45.5%	3	60.0%	12	36.4%	15	50.0%	0.63
Personal or family history of MRSA	11	5.7%	3	3.6%	1	14.3%	3	5.8%	4	8.2%	0.53
Comorbidities	10	5.2%	2	2.4%	0	0.0%	4	7.7%	4	8.2%	0.35
Ultrasound	104	54.2%	45	53.6%	3	42.9%	27	51.9%	29	59.2%	0.81
Cellulitis	106	55.2%	49	94.2%	3	75.0%	22	95.7%	32	100.0%	0.13
Fluctuance	80	41.7%	31	56.4%	4	66.7%	23	67.6%	22	71.0%	0.53
Wound Culture	82	42.7%	30	35.7%	4	57.1%	22	42.3%	26	54.2%	0.18
Blood Culture	41	21.4%	16	19.0%	1	14.3%	9	17.3%	15	30.6%	0.32
I&D	105	64.8%	43	51.2%	4	57.1%	28	53.8%	30	61.2%	0.73
I&D in OR	27	14.1%	9	16.1%	1	25.0%	10	32.3%	7	24.1%	0.54
Procedural Sedation	43	22.4%	15	34.1%	3	100.0%	9	42.9%	16	64.0%	0.03
IV Antibiotics	68	35.4%	24	28.6%	2	28.6%	17	32.7%	25	51.0%	0.06
Oral Antibiotics	42	21.9%	17	20.2%	0	0.0%	10	19.2%	15	30.6%	0.21
Admitted	54	28.1%	18	21.4%	2	28.6%	15	28.8%	19	38.8%	0.20
Followed-Up	29	15.1%	12	100.0%	1	100.0%	8	100.0%	8	88.9%	0.49
Recurrence	11	5.7%	3	9.1%	1	25.0%	5	26.3%	2	7.1%	0.19

WBC, white blood cell count; CRP, c-reactive protein; MRSA, methicillin-resistant *Staphylococcus aureus*; I&D, incision and drainage; OR, operating room; IV, intravenous
